# Successful local and systemic medical treatment of cesarean scar pregnancy and a subsequent term pregnancy after treatment: a case series

**Published:** 2015-07

**Authors:** Reihaneh Pirjani, Leila Bayani, Mahboobeh Shirazi

**Affiliations:** 1 *Department of Obstetrics and Gynecology, Arash Hospital, Tehran University of Medical Sciences, Tehran, Iran.*; 2 *Department of Radiology Arash Hospital, Tehran University of Medical Sciences, Tehran, Iran.*; 3 *Maternal Fetal and Neonatal Research Center, Tehran University of Medical Sciences, Tehran, Iran.*

**Keywords:** *Cesarean*, *Ectopic pregnancy*, *Potassium chloride*, *methotrexate*

## Abstract

**Background:**

Treatment of cesarean scar pregnancy (CSP) is controversial. The objective of this study was to report our successful experience in the medical treatment of CSP with potassium chloride (KCl) and methotrexate.

**Case:**

This is a case series of six patients between 6-12 gestational weeks with the diagnosis of CSP. In five cases the fetus was alive and in one case, despite being at a gestational age of 12 weeks based on CRL, there was no fetal heart activity. In four of these cases, an ultrasound- guided KCl injection in the heart was performed on four living fetuses and then systemic methotrexate was administered. In two other cases, methotrexate was injected into the gestational sac and subsequently the systemic methotrexate was administered. During follow-up, the patients were stable and no complications occurred. Additionally, serum beta human chorionic gonadotropin (β-hCG) was negative between five to 11 weeks later. One of the patients became pregnant one year later. Her pregnancy continued without any complication and she was delivered by cesarean section at the gestational age of 38 weeks. During caesarean section, it was noticed that the appearance of previous cesarean scar was normal and there was no scar.

**Conclusion:**

Based on our experience, the combination of systemic Methotrexate with local Methotrexate or KCl is feasible and can be performed as an outpatient procedure and is successful in the treatment of CSP.

## Introduction

Cesarean scar pregnancy (CSP) is a relatively rare type of pregnancy. The incidence of CSP has been reported between 1/1800 to 1/2216 pregnancies (1). CSP may be associated with severe complications and may carry a life threatening condition due to the possibility of uterine rapture, invasive placenta and severe bleeding. Increasing rate of CSP during the last two decades necessitates a proper treatment of CSP, but there is no consensus on optimal treatment. In this paper we report our experience in the medical treatment of six women with viable CSP and subsequent pregnancy outcome in one of them.

## Case Series

**Figure 1:**
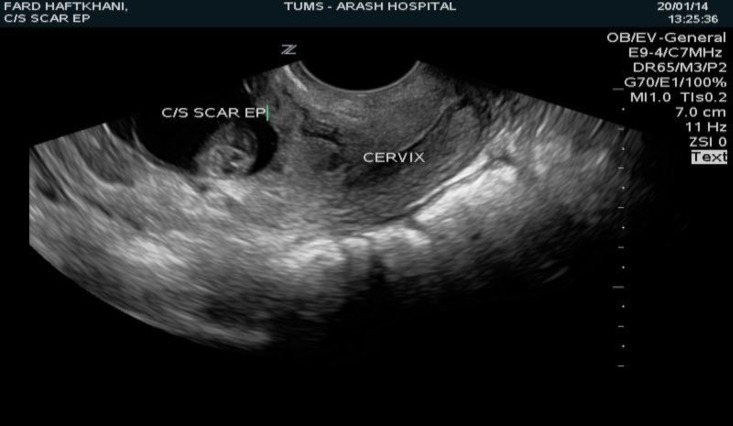
Cesarean scar pregnancy demonstrated by transvaginal sonography showing the gestational sac implanted in the lower segment of the uterus

**Figure 2:**
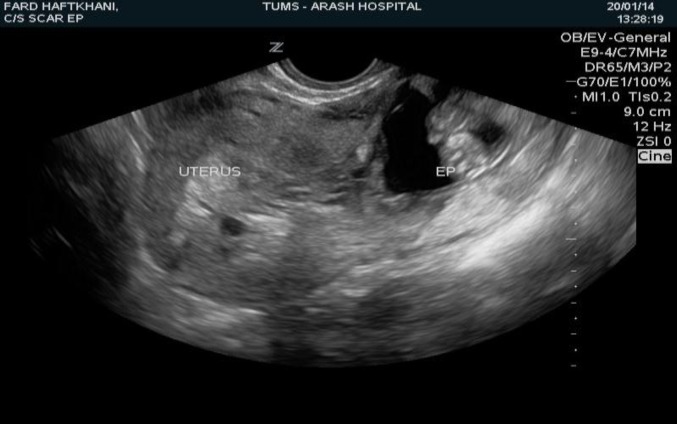
Transvaginal ultrasonography showing an empty uterine cavity with the gestational sac containing a fetus alive in the lower segment of the uterus (Cesarean scar pregnancy)

In this Case Series study CSP was diagnosed on the basis of the following criteria described by Wang et al (2): An empty uterus and cervical canal, with a clearly demonstrated endometrium; a gestational sac located at the anterior wall of the uterine isthmic portion surrounded by the myometrium and fibrous tissue of the scar and separated from the endometrial cavity or fallopian tube. (Figures 1, 2).

Written consent was obtained from all paiteints.

**Case 1:** A 29-year-old woman with history of two previous curettages and one cesarean section was referred for first trimester screening. Ultrasonography was performed and the gestational sac with a live fetus was seen near the bladder. Gestational age was 11 weeks and five days based on CRL. After confirming cesarean scar pregnancy with abdominal and vaginal probe, the patient was counseled regarding her management options, and she had opted for medical treatment. She had a normal full blood count and liver/renal function test and her vital signs were stable. Abdominal ultrasound-guided potassium chloride (KCL) (2.5 cc KCl 50%) was injected into the fetal heart and fetal death occurred.

Serum level of beta-human chorionic gonadotrophin (β-hCG) was 53173 IU/ml before KCL injection and reduced to 45128 IU/ml after 24 hours. Progressive loss of β-hCG titer continued so that 14 days after injection, the level of serum β-hCG was 5708 IU/mL. During this period, the patient's vital signs were stable and no bleeding and no complications occurred. Despite the appropriate decrease in β-hCG titer, the pregnancy mass was massive (56 x 80 mm) and thus two doses of methotrexate (50mg/m2) were administered 14 and 21 days after KCl injection, to prevent possible bleeding. Blood level of β-hCG was negative nine weeks after starting treatment, and finally four months after starting treatment, the pregnancy mass completely absorbed. During treatment, no side effects were observed. A year later the patient became pregnant. Pregnancy continued without any complication and she was delivered by cesarean section at the gestational age of 38 weeks. During caesarean section it was found that the appearance of previous cesarean scar was normal and there was no scar.

**Case 2:** A 28-yea-old woman, gravida four, para two, abort one was referred to our center after spotting and abdominal pain. She had two previous ultrasounds at six weeks and nine weeks of pregnancy that showed gestational sac with a living fetus. She had a history of two previous cesarean sections and a previous curettage. Vaginal sonograghic examination revealed an empty endometrial cavity and a gestational sac containing an alive fetus between uterine and bladder surrounding by myometrium. Gestational age was nine weeks and five days. She had a normal full blood count and liver/renal function test and her vital signs were stable. Serum β-hCG titer was 104898 IU/ml. After informed consent, 2.5 ml KCl 50% was injected into the fetal heart abdominal ultrasound-guided and then she received a course of treatment with methotrexate (one mg/kg for four doses) and leucovorin (0.1 mg/kg for four doses). One day after KCl injection, serum levels of β-hCG titer fell to 94003 IU/ml. Serum β-hCG was negative eight weeks later and the mass was not visible with ultrasound five months later.

**Case 3:** The patient was a 42-year old woman, gravida four, para two, abort one who had a history of two previous cesarean sections and a previous curettage. She was referred to our center with a diagnosis of cesarean scar ectopic pregnancy. Physical examination was negative and she had no symptoms. Gestational age was seven weeks and five days, the fetus was alive and serum level of β-hCG was 40413 IU/ml. After confirming cesarean scar pregnancy and obtaining informed consent from the patient, she underwent injection of 2.5 ml Kcl 50% into the gestational sac using transvaginal ultrasound-guide. Two doses of methotrexate (50mg/m2) were administered on one and seven days after injection of KCl. The next day β-hCG level increased to 49283 IU/mL and decreased to 18729 7 days after KCl injection. During follow-up, the patient was stable and no complications occurred. Serum β-hCG was negative 11 weeks later. Four months later the patient had a regular menstrual cycle but an echogenic mass as high as 10 to 12 mm was still presented on sonography six months later.

**Case 4:** A 34-year-old woman with a history of two previous cesarean section and two previous miscarriages was referred to our hospital. She had an ultrasound that showed an empty uterus with a gestational sac between uterus and bladder. Transvaginal sonography was performed in our center and CSP was confirmed. Gestational age was 13 weeks based on LMP and 12 weeks based on CRL. There was no fetal heart activity and a 71×59 mm hematoma was found around the gestational sac. She had mild abdominal pain, but her vital signs were stable. Her general condition was good and she had a normal, full blood count and liver/renal function test. She was hospitalized and after obtaining informed consent, ultrasound-guided methotrexate injection (60 mg) was performed into the gestational sac. The level of β-hCG on the same day was 874 IU/mL and fell to 329 IU/mL 7 days later. She was hospitalized for a week and during this time received four doses of methotrexate (one mg/kg/Im) and four doses of leucovorin (0.1 mg/kg/Iim). During follow-up, she was stable and no complications happened. Serum β-hCG was negative eight weeks later and the mass was not visible with ultrasound three months later.

Case five: A 37-year-old woman with a history of one previous cesarean section and one previous curettage was presented with complaints of spotting. Serum level of β-hCG was 39403 IU/mL. Transvaginal sonography was performed in our center and revealed CSP. Gestational age was eight weeks based on CRL and the fetus was alive. She had a normal full blood count and liver/renal function test and her vital signs were stable. Informed consent was obtained and 2.5 ml Kcl 50% was injected into the gestational sac. The level of β-hCG on day seven after Kcl injection was 24661 IU/mL. One week after discharge, vaginal bleeding occurred that was not severe and did not require intervention. Serum β-hCG was negative eight weeks later and four months after starting treatment, the pregnancy mass completely absorbed.

Case six: A 40-year-old woman with a history of two previous cesarean sections and one curettage was referred for prenatal care at 6 weeks of gestation. We performed a pregnancy routine ultrasound and during this exam, the presence of CSP was noticed. Gestational age was six weeks based on CRL, fetal heart activity was presented and serum level of β-hCG was 5321 IU/ml. Informed consent was obtained and one dose of systemic methotrexate (50mg/m2) was administered. Two days later, methotrexate (60 mg) was injected into the gestational sac. During follow-up, no complication occurred and five weeks later, serum level of β-hCG was negative.

**Table I:** Clinical summary of six cesarean scar pregnancy cases

	**Case one**	**Case two**	**Case three**	**Case four**	**Case five**	**Case six**
Gestational age at diagnosis (weeks)	11weeks	9 weeks	7weeks	12 weeks	7 weeks	6weeks
Fetal heart beat	+	+	+	─	+	+
Initial β-hCG level (IU/ml)	53173	104898	40413	874	39403	5321
Initial treatment	Local KCl	Local KCl	Local KCl	Local MTX	Local KCl	Systemic MTX
Second treatment	Systemic MTX	Systemic MTX	Systemic MTX	Systemic MTX	Systemic MTX	Local MTX
Number of previous cesarean	one	two	two	two	one	two
History of previous curettage	+	+	+	+	+	+
Time to reach nondetectable β-hCG level (weeks)	nine	eight	11	eight	eight	five
Time to disappearance of CSP mass(months)	four	five	10×12 mm mass remained	three	four	three
Side effect and bleeding during treatment	none	none	none	none	none	none

β-hCG: Beta human chorionic gonadotropin

CSP: Cesarean scar pregnancy

## Discussion

In recent years, many cases of CSP have been reported and various therapies have been introduced. The pathophysiology of CSP is not clearly known. The conceptus may infiltrate through a microscopic dehiscent tract of the myometrium and grow (3). In CSP, fertilization is normal but implantation occurs at the lesion site and bulges from the uterine wall in the cesarean scar (4). In addition, it can be assumed that blastocyst invades to any microscopic lesion, due to other causes such as myomectomy, hysteroscopy and even manual removal of the placenta (5). All of our patients had a previous history of curettage supporting this supposition.

Although the first case of CSP was reported in 1978 and only about 40 case reports and series have been reported up until 2006, within the past three years further studies on CSP have been published, that indicate an increase in CSP incidences. CSP incidences varied among studies (6-11), for example, Fadhlaoui et al were faced with only one case of CSP during a time frame of one year at their department (one of the total 62 ectopic pregnancies) (12). However, Wang, et al. had 71 CSP cases in two years at their hospital (13). They did not mention the incidence of cesarean section and ectopic pregnancy in their hospital, but it appears that the CSP incidences were high at their hospital. We do not know the number of CSP incidences in Iran. Unfortunately, in Iran, the cesarean section rate is high and so we will be faced with more cases of CSP in the future.

Different treatment modalities are used in the management of CSP. Some authors consider surgical methods such as laparoscopy and hysteroscopy (14, 15). Some others make use of medical treatment such as local or systemic MTX or local KCl (16, 17). Wang, et al introduced a new transvaginal surgical approach for CSP (18). Another treatment modality is uterine artery embolization (UAE), which can be used alone or in combination with other methods (19, 20). Some authors proposed a combination of different methods (21, 22). Tulpin, et al. reported a case of CSP, where they injected MTX into the gestational sac during laparoscopy (23). UAE was then performed three times up to 24 days after laparoscopy. To put differently, they applied medical treatment using minimally invasive laparoscopic technique. Timor-Tritsch and. Monteagudo published a review article on the consequences of cesarean delivery (9). They concentrated on placenta previa and CSP. Their research concluded that local MTX and hysteroscopic directed procedure resulted in the lowest level of complications, while in contrast to that, curettage, systemic MTX and UAE as single treatment should be avoided if possible (9).

One hypothesis is that the likelihood of complications and failure may be related to gestational age. Cignini and coworkers (5) reported two cases of CSP, of which one case was at nine gestational weeks and underwent laparotomy. Subtotal hysterectomy was performed duo to severe hemorrhage. Another case was reported at sixth week of pregnancy, which underwent hysteroscopy and the treatment resulted in a success. They concluded that early detection of CSP seemed to reduce complications. It is clear that early detection of CSP will lead to a reduced risk of bleeding and other complications. However, in terms of responsiveness to treatment, our study showed that medical treatment would be successful even at higher gestational age. In case #1, pregnancy was at an advanced stage (12 weeks) and after a successful medical treatment, the patient had a term subsequent pregnancy. Based on our experience, it seems unlikely that serum levels of β-hCG influence the response to treatment.

In our opinion, local MTX or KCl therapy in combination with systemic MTX prove to be an effective method for treatment of CSP. One of the advantages of this method is that it is outpatient basis and does not require hospitalization. Another advantage is that patients could avoid the risks of surgery and anesthesia-related complications. In contrast, the disadvantage of this method is that it takes several weeks to be negative beta-HCG and several months to be indistinguishable CSP mass by ultrasound.

In conclusion, the management of CSP is not well established and there is no consensus about its treatment. However, given our experience and findings, the combination of systemic MTX with local MTX or KCl administration is feasible and can be performed as an outpatient procedure, which will hopefully lead to a successful treatment of CSP.
